# From “Gig” to Genes: The enduring legacy of Seymour Levine in developmental stress neurobiology

**DOI:** 10.1016/j.ynstr.2025.100772

**Published:** 2025-11-26

**Authors:** Mathias V. Schmidt

**Affiliations:** Research Group Neurobiology of Stress Resilience, Max Planck Institute of Psychiatry, Munich, Germany

## Abstract

The concept that early life experiences profoundly program adult physiology and behavior is now a cornerstone of neurobiology, a paradigm shift largely founded on the visionary work of Seymour “Gig” Levine. This review traces the intellectual lineage from Levine's pioneering behavioral and physiological studies to the modern molecular era, underscoring his indispensable contribution to understanding developmental stress. Levine's initial elegant experiments established the critical regulatory role of maternal care and the hypothalamic-pituitary-adrenal (HPA) axis, culminating in the discovery of the Stress Hyporesponsive Period (SHRP)—a crucial, sensitive window for developmental programming. Building on this legacy, subsequent research, including my own, has utilized advanced molecular tools to bridge the gap from these macroscopic observations to precise mechanistic detail. I highlight the role of gene-by-environment (G × E) interactions, particularly involving HPA axis modulators like FKBP51 and CRH signaling, in shaping vulnerability. Furthermore, I discuss how Levine's implicit recognition of individual differences has evolved into a central focus on biological sex differences, the match/mismatch hypothesis of adaptive programming, and the use of deep phenotyping to unravel the molecular bases of resilience and vulnerability. Ultimately, the journey from “Gig” to “Genes” provides a foundational understanding that is crucial for developing effective, targeted strategies to promote mental health and resilience across the lifespan.

## 1 Introduction

Our current understanding that experiences early in life can significantly shape later-life physiology and behavior is remarkably recent. For a considerable period, an individual's behavioral and physiological properties were widely considered to be largely fixed at birth and determined primarily by genetics. While the enduring “nature versus nurture” discussion certainly existed, it was historically heavily weighted towards the “nature” component (Cowan, 1977; McEwen, 1988; Rutter et al., 1997). Sigmund Freud, in the late 19th and early 20th centuries, was arguably one of the first to popularize the idea that early childhood experiences have a profound and lasting impact on adult personality and psychological well-being, though his insights were largely based on clinical observation rather than empirical experimentation (Freud, 1938). A significant shift occurred in the 1950s and 60s, notably with Harry Harlow's groundbreaking work with Rhesus Macaques. Harlow's experiments compellingly demonstrated the critical importance of emotional and social factors in early development, proving their profound influence extended far beyond simple biological needs (Harlow and Suomi, 1970). However, Harlow's and others' work implicitly raised crucial questions about the underlying biological mechanisms responsible for these observed long-term effects of the early life environment. Specifically, how did fundamental influences such as maternal contact physiologically influence development in such a lasting manner? This pivotal question, which ultimately laid the foundation for our contemporary understanding of the influence of early life stress and trauma, was meticulously tackled by Seymour “Gig” Levine. His enduring legacy continues to form the bedrock of our understanding of these complex biological mechanisms today.

My personal connection to this field began when I first met Gig (his much-preferred nickname in all personal exchanges) during an exchange semester at the University of Delaware in 1998, where I intended to study animal physiology. I discovered him in his cigarette fume-filled office—a striking contrast to the otherwise strictly non-smoking environment—and was soon adopted into his scientific family. This “adoption” encompassed not only joint fishing trips, sports events, and fabulous home dinners but, most importantly, the passionate discussion and rigorous study of how early life influences shape adult physiology and behavior. Throughout his memorable career, Levine laid the fundamental groundwork for the insight that early life experiences are not merely transient events but profoundly program an organism's long-term stress reactivity and behavior. He rigorously demonstrated that this programming occurs through the central role of the hypothalamic-pituitary-adrenal (HPA) axis and its critical involvement in mediating the stress response during specific, sensitive windows of development. The scope of this review is to trace the intellectual lineage from Levine's pioneering behavioral and physiological studies to the modern era of molecular neurobiology, highlighting the subsequent contributions that have unraveled the genetic and epigenetic underpinnings of developmental stress programming and their profound consequences for health and disease across the lifespan.

## 2 Seymour “Gig” Levine's legacy: pioneering behavioral and physiological insights to developmental neurobiology

Levine's initial studies, often employing simple yet elegant experimental paradigms, involved daily brief periods of handling (e.g., 3–15 min of separation from the mother) of rat pups during their first few weeks of life. These seemingly minor interventions yielded remarkably robust and long-lasting effects on the offspring's HPA axis, the primary neuroendocrine system mediating the stress response, and their subsequent behavior (Levine, 1957; Meaney et al., 1991). When exposed to novel stressors in adulthood (e.g., restraint, novel environment), handled rats showed a significantly blunted and more rapid termination of their corticosterone (the primary glucocorticoid in rodents) response compared to non-handled controls (Levine, 1970). This indicated a more efficient and less exaggerated physiological stress response. On a behavioral level, handled animals often displayed more adaptive behavioral responses to stress. They were typically less fearful of novelty, more explorative in new environments, and showed reduced anxiety-like behaviors in paradigms such as the elevated plus-maze (Cirulli et al., 2010). Some studies also indicated that early handling could enhance cognitive functions, particularly those related to stress-sensitive learning and memory tasks. Subsequent research, building on Levine's initial observations, elucidated the neurobiological underpinnings. Handled rats showed differences in glucocorticoid receptor (GR) expression in key brain regions like the hippocampus and prefrontal cortex, as well as alterations in neurotransmitter systems (e.g., serotonin, GABA) involved in stress regulation (Meaney et al., 1996, 2013). These neurochemical changes contributed to the observed HPA axis and behavioral phenotypes. Crucially, Levine and his colleagues demonstrated that these effects were not simply due to the brief stress of separation during handling, but rather to the maternal reunion and increased maternal licking and grooming that often followed the handling procedure (Hennessy et al., 1980). The handled pups, upon return to the nest, elicited more maternal care from their mothers, and it was this enriched maternal interaction that was later identified as the primary mediator of the beneficial effects. This shifted the focus from the handling itself to the quality and quantity of maternal care.

Beyond demonstrating the general impact of early experience, Levine's group was instrumental in identifying “critical periods” during which the developing HPA axis is particularly sensitive to environmental input. One of the most significant discoveries in this regard was the Stress Hyporesponsive Period (SHRP) (Levine et al., 2000). The SHRP refers to a specific developmental window in rodents (roughly postnatal days 4–14 in rats; postnatal days 2–12 in mice) during which the HPA axis exhibits a markedly attenuated response to novel stressors (Schmidt et al., 2005). During this period, pups show minimal or no increase in corticosterone levels even when exposed to stimuli that would elicit a robust response in older or younger animals. The presence of the mother is critical for maintaining this hyporesponsiveness. Maternal deprivation during the SHRP can disrupt this period of attenuated reactivity, leading to an exaggerated HPA response (Suchecki et al., 1993). This highlighted the mother's role as a primary regulator of the pup's stress physiology. While seemingly a period of protection, the SHRP is paradoxically a time of immense vulnerability for developmental programming. Disruptions to the maternal environment during this window (e.g., prolonged maternal separation, exposure to unpredictable maternal care) can permanently alter HPA axis set points, leading to hyper-responsive stress systems and increased vulnerability to stress-related disorders in adulthood. Conversely, positive maternal interactions during this period can “program” a more resilient stress response. The concept of the SHRP underscored that the timing of environmental events is as crucial as their nature. The developing HPA axis is not uniformly plastic but rather undergoes phases of heightened sensitivity, during which specific experiences can exert disproportionate and lasting effects on its structure and function. Levine's work fundamentally challenged existing dogma, inspiring generations of researchers (including myself) to meticulously explore the “how” and “why” of the profound and lasting consequences of early life challenges on later behavior and physiology.

## 3 Bridging the gap: from behavioral observations to biological mechanisms

The profound observations made by Seymour Levine and others on the lasting impact of the early life environment on stress reactivity and behavior gained significant relevance in the context of stress-related psychiatric disorders. This recognition fueled a critical need for understanding the precise cellular and molecular machinery underlying these enduring effects. Fortunately, this deeper investigation was made possible by the rapid emergence of sophisticated molecular tools and transformative advancements in molecular biology and neuroscience. These innovations allowed researchers to move beyond behavioral observations and delve into the intricate biological underpinnings of developmental programming. Key advancements included large-scale gene sequencing, the burgeoning field of epigenetics (which examines heritable changes in gene expression not caused by changes in the DNA sequence), advanced imaging techniques, and the development of powerful mouse genetic tools. Furthermore, the advent of precise circuit manipulations through technologies like optogenetics or DREADDs (Designer Receptors Exclusively Activated by Designer Drugs) provided unprecedented control over neural activity, enabling direct investigation into the brain circuits mediating these long-term effects.

An important aspect that became increasingly clear was the significant individual variability in response to early life adversity (Boyce et al., 2021; Daskalakis et al., 2013; Hartmann and Schmidt, 2020). Not all individuals exposed to similar early life challenges are impacted at the same level; some exhibit profound vulnerabilities, while others demonstrate remarkable resilience. This observation naturally led to the critical question of resilience itself: can we understand and consequently predict why some individuals are more vulnerable to stress perturbations, while in others, stress exposure paradoxically strengthens their resilience? Thus, Levine's foundational work directly influenced this shift in research direction, guiding the focus towards unraveling the molecular consequences of developmental stress and the mechanisms of both vulnerability and resilience.

## 4 Elucidating molecular and (epi)genetic mechanisms of early life adversity

The profound importance of gene-by-environment (G × E) interactions in shaping psychopathology following early life stress was underscored by clinical studies in the early 2000s, which demonstrated that functional polymorphisms in stress-relevant genes profoundly impact disease risk (Binder et al., 2008; Bradley et al., 2008; Caspi et al., 2003; Caspi and Moffitt, 2006). This pioneering work fueled subsequent investigations into the differential consequences of ELS dependent on underlying genetic risk factors, particularly those involved in HPA axis regulation (Yam et al., 2015). Early research established the critical role of GR feedback mechanisms in mediating ELS effects (Meaney et al., 1993). Subsequently, modulators of GR function, specifically the co-chaperone FKBP51, were implicated in robust G × E interactions related to stress resilience and susceptibility (Binder, 2017; Matosin et al., 2018). Recently, our lab further defined the crucial role of FKBP51 signaling in mediating the downstream effects of ELS exposure (van Doeselaar et al., 2025), which was cell-type and sex specific. Another HPA axis mediator that was heavily implicated in the consequences of early life adversity was corticotropin-releasing hormone (CRH). We first showed that in mice, CRH is dynamically regulated during the postnatal stress hyporesponsive period (SHRP) and following early life stress (Schmidt et al., 2003, 2004). This foundational finding was built upon by our group and others, demonstrating that the brain CRH-CRHR1 system is causally involved in the long-term emotional and behavioral consequences of ELS (Brunson et al., 2005; Schmidt et al., 2006), positioning CRH as a central driver of HPA axis-ELS interactions (Liao et al., 2014; Wang et al., 2011, 2012, 2013; Yang et al., 2015). However, these G × E interactions do not act in isolation, but rather in concert with a wide variety of biological processes that are programmed by early life adversity (Malave et al., 2022), including complex stress interactions with metabolism (Yam et al., 2015) and the immune system (Cattane et al., 2022). Underlying this lasting programming is epigenetic regulation, which provides a key mechanism for long-term transcriptional changes induced by ELS (Peña, 2025). The role of epigenetics in ELS has been extensively reviewed, focusing on mechanisms like DNA methylation and histone modifications (Alyamani and Murgatroyd, 2018; Bertollo et al., 2024; Provençal and Binder, 2015).

These molecular mechanisms frequently exhibit a highly brain-region-specific action. Initial studies concentrated on the hippocampus, a brain region central to stress pathology due to its high expression of mineralocorticoid receptors (MR) and GR, and its integral role in emotional regulation and memory. Early life stress profoundly determines the long-term effects of glucocorticoids and stress on hippocampal function (Pillai et al., 2018). We have specifically shown that hippocampal cell adhesion molecules, such as neuroligin, are key contributors to the enduring effects of ELS (Kohl et al., 2015). Similar effects have been observed in hippocampal neurogenesis (Korosi et al., 2012) and synaptic plasticity, mediated by factors like brain-derived neurotrophic factor (BDNF) (Roth et al., 2009). The impact of ELS on hippocampal function and memory has been recently and thoroughly summarized (Birnie and Baram, 2025). Advancements in single-cell transcriptomics have further refined this understanding, identifying hippocampal GABA-ergic signaling as a critical mediator of ELS consequences (Kos et al., 2023). Crucially, ELS affects not only neuronal populations but also results in long-term plasticity in resident glial cells, including alterations in oligodendrocytes (Teissier et al., 2020), microglia (Reemst et al., 2022), and astrocytes (Çalışkan et al., 2020), underscoring the widespread cellular impact of early life adversity. These examples represent only a fraction of the mechanistic insights gathered over the last decades, but collectively highlight the enduring conceptual and intellectual contribution of Seymour Levine's foundational work in the field of early life stress.

## 5 Resilience and vulnerability to early life stress

In a review in 2010, I already stated: “Specific genetic or epigenetic predispositions influence the adaptive changes to early life stress, which are generally maladaptive in our modern society. It is therefore clear that any animal model that does not account for a proportion of resilient individuals will also fall short in explaining the molecular basis of early life stress-induced effects. Unfortunately, there are very few reports that address this critical issue using an unbiased approach.” (Schmidt, 2010). In fact, Levine's work already established the foundation for studying resilience and individual variability, long before those terms became commonplace in the molecular neurobiology field (Levine, 2005). Since then, the field has gained significant momentum, elucidating many aspects of how early life experiences do not always lead to pathology. One central determinant of differential outcomes that has been recognized is biological sex. While the majority of research in the era of Levine focused on males—with females often disregarded—we now understand that biological sex is a prime determinant of the response to early adversity, with vulnerability and resilience to future stress governed by diverging molecular and circuit mechanisms in males and females (Bordes et al., 2024). Furthermore, building conceptually on Levine's insights regarding early programming, the match/mismatch hypothesis was proposed: This hypothesis posits that a phenotype programmed by early adversity can be adaptive if the adult environment 'matches' the expected stressful environment, but maladaptive if the adult environment 'mismatches' the early-life prediction (Nederhof and Schmidt, 2012). Thus, moderate early life adversity can context specifically indeed lead to increased stress resilience. This principle has now been confirmed numerous times in both animal models (Santarelli et al., 2017) and human cohorts (Hoogland and Ploeger, 2022; Nederhof, 2013). Furthermore, we have also started to understand that proposed genetic risk factors, such as FKBP51, can also act as resilience factors in specific contexts, for example by enabling stress-mediated plasticity and adaptation rather than simply increasing susceptibility (van Doeselaar et al., 2025). Finally, the study of true individual stress vulnerability and resilience in animals is now possible with recent advances in machine-learning assisted deep phenotyping (Bordes et al., 2023; Mathis et al., 2018), which enables the long-term, unbiased observation of complex behavioral traits in freely moving mice (e.g., home cage monitoring). Taken together, modern research on sex differences, adaptive programming, and individual variability moves beyond a simple definition of ELS-induced pathology to fully embrace the heterogeneity of response, a phenomenon implicitly recognized by Levine's earliest observations on differential outcomes.

## 6 Conclusion: the enduring echo of “Gig” in the era of “Genes"

This review traces the indispensable intellectual lineage from Seymour “Gig” Levine's pioneering work in the mid-20th century to the sophisticated molecular neurobiology of today. Levine's visionary experiments, which established the profound and lasting impact of early experiences on the hypothalamic-pituitary-adrenal (HPA) axis and behavioral outcomes, provided the fundamental conceptual framework for the entire field of developmental stress neurobiology. His legacy is the foundation upon which subsequent generations of researchers, including our own lab, have meticulously built, successfully bridging the gap from macroscopic behavioral observations to precise, cell-type-specific molecular and epigenetic mechanisms. The detailed understanding of gene-by-environment interactions, sex differences in vulnerability, and the intricate dynamics of resilience and adaptive programming are all direct continuations of the questions Levine first posed. Ultimately, the knowledge gained from this journey—from “Gig” to “Genes"—is not merely academic; it is crucial for developing effective, targeted strategies that can interrupt the cycle of developmental stress and promote mental health and resilience across the human lifespan. The principles that Levine established—that early care can program adult physiology and that timing is paramount—continue to resonate, ensuring that his influence remains the bedrock of developmental research for decades to come.

## Declaration of generative AI and AI-assisted technologies in the manuscript preparation process

During the preparation of this work the author used Gemini version 2.5Flash, a large language model built by Google, for language editing. After using this tool/service, the author reviewed and edited the content as needed and takes full responsibility for the content of the published article.

## Declaration of competing interest

The author declares no financial or professional conflicts of interest regarding the submitted work. The manuscript addresses the legacy and contributions of the late Dr. Seymour Levine to the field of psychobiology. The author was a former student of Dr. Levine, which may introduce a non-financial, intellectual bias toward the subject matter. This potential bias has been noted and every effort has been made to ensure a critical and objective presentation of the material.

## Data Availability

No data was used for the research described in the article.
